# Fitting the reproduction number from UK coronavirus case data and why it is close to 1

**DOI:** 10.1098/rsta.2021.0301

**Published:** 2022-10-03

**Authors:** Graeme J. Ackland, James A. Ackland, Mario Antonioletti, David J. Wallace

**Affiliations:** ^1^ School of Physics and Astronomy, Edinburgh EH9 3FD, UK; ^2^ EPCC, University of Edinburgh, Edinburgh EH9 3FD, UK; ^3^ Department of Psychology, University of Cambridge,Cambridge CB2 3EB, UK; ^4^ University of St Andrews, St Andrews, Fife, UK

**Keywords:** coronavirus, *R*-number, epidemic, SIR model, compartment model

## Abstract

We present a method for rapid calculation of coronavirus growth rates and R-numbers tailored to publicly available UK data. We assume that the case data comprise a smooth, underlying trend which is differentiable, plus systematic errors and a non-differentiable noise term, and use bespoke data processing to remove systematic errors and noise. The approach is designed to prioritize up-to-date estimates. Our method is validated against published consensus R-numbers from the UK government and is shown to produce comparable results two weeks earlier. The case-driven approach is combined with weight–shift–scale methods to monitor trends in the epidemic and for medium-term predictions. Using case-fatality ratios, we create a narrative for trends in the UK epidemic: increased infectiousness of the B1.117 (Alpha) variant, and the effectiveness of vaccination in reducing severity of infection. For longer-term future scenarios, we base future R(t) on insight from localized spread models, which show R(t) going asymptotically to 1 after a transient, regardless of how large the R transient is. This accords with short-lived peaks observed in case data. These cannot be explained by a well-mixed model and are suggestive of spread on a localized network.

This article is part of the theme issue ‘Technical challenges of modelling real-life epidemics and examples of overcoming these’.

## 1. Introduction

During the coronavirus epidemic, the so-called ‘R-number’ has become one of the best-known concepts from epidemiology. It can be defined as *the average number of onward infections from each infected person.* It is conventional to define $R_{0}$ as the R-number at the outset of an outbreak, and $R_{t}$ as its value some time t later. The significant feature is that an R-number greater than 1 implies an exponential growth in case numbers, whereas an *R*-number less than 1 implies exponential decay. Typically, $R_{t}<R_{0}$ due to acquired immunity or behavioural changes reducing spread. The *R-*number is often used by policymakers to trigger interventions. It is particularly useful because it is a leading indicator: it can been seen to exceed the epidemic value of *R* = 1 long before cases, hospitalizations and deaths reach critical levels. Local measures of the *R*-number enable governments to use well-focused interventions to achieve maximum disease suppression with minimal disruption. However, care must be taken that the correct measure is being used.

### (a) Defining R

In a real epidemic, this conceptual definition of $R_{t}$ is ambiguous: it may refer to people infected at time t, or to people infectious at time t, or to the rate of infection at time t.^1^ The first two definitions incorporate infections in the future, and therefore under these definitions $R_{t}$ is unknowable at time t. If using the third definition, the conversion from growth rate to R depends on some model for how the epidemic is spreading, such as ‘exponential growth’, which is generally true only for a homogeneous, well-mixed population. If the outbreak is spreading in space, then infectors may come from a different population from the infectees, and the epidemic will be limited by diffusion so that cases will not grow exponentially.^2^

Further ambiguity comes from the term ‘average’. Clearly, each infected individual is responsible for an integer number of onward infections, and one can extend the idea to the number of onward infections that an individual would make if they were infected. Then the average might be taken over the whole population, which would give the R representing risk of the infection becoming epidemic. Or, the average could be taken only over those who are actually infected, in which case R represents the current growth rate. In an inhomogeneous population, these measures are likely to be different because early victims will come preferentially from those with many contacts (i.e. high individual R).

Any epidemic model which does not represent each individual person cannot simply count the number of subsequent infections per person. Thus definitions of R are usually related to growth rate. Assuming that the number of new infections is proportional to the number of currently infected people, *I*(*t*), the growth rate is

1.1 $$\frac{dI(t)}{dt}/I(t)=\frac{d\ln I(t)}{dt}=[(R_{t}-1)/\tau ],$$

which introduces a timescale τ, similar to the time between infections and therefore referred to as the generation time^3^ [1–3]. This definition of $R_{t}$ shares important features with other definitions, in particular that R=1 is the critical value separating a growing and diminishing outbreak. Its advantage is that it does not depend on future events.

Our approach to R is even more pragmatic. We define R as a quantity based directly on available data which satisfies the constraint that R=1 is the critical value and reproduces the rate of growth of the epidemic. In practice, this means using the equivalent to equation (1.1) based on reported positive PCR test data, i.e. number of cases, C(t), in place of infection data. This leads to a different $R_{t}$, defined by

1.2 $$\frac{dC(t)}{dt}/C(t)=\frac{d\ln C(t)}{dt}=\left[(R_{t}-1)/\tau \right].$$

Now τ is the time lag between the infector and infectee showing symptoms, sometimes known as the serial interval. Not all infections will be reported, and reported cases may include false positives. A vital feature of this equation is that even if only a fraction of infections is reported, that fraction cancels out: $R_{t}$ is independent of systematic underreporting; for example, if there is a fixed fraction of asymptomatic and infectious individuals who are never reported in C(t), the R calculation is still correct. The prevalence of the disease will also be incorrect, but this can be corrected for with an ‘undetected case’ fraction, which is evaluated using data from the Office for National Statistics (ONS) prevalence survey [4]. This fraction is substantial for COVID-19—it appears that most cases are never reported—however, the discrepancy between ONS incidence data and reported data remained constant over time until the removal of PCR testing in early 2022.

Equation (1.2) is surprisingly insensitive to reporting changes over time: reporting is a behavioural issue and in 2020–2021 it varied slowly on the generation timescale. In February 2022, the UK had a policy change to discontinue PCR confirmation tests, which led to a step change in the published reported cases. Even this has little effect because, being instantaneous, it only affects the change in cases on one, known, day. This one-day change (in practice occurring over a few days) can be eliminated from the data, and log differences log⁡[C(t+1)/C(t)] on either side of the abrupt change are unaffected. Thus any reporting changes on a timescale different from the generation time, either much longer or much shorter, do not affect the reliability of the R-number estimate. This provides greater stability to our discrete-time kernel model than can be achieved with a Bayesian fit to a set of ordinary differential equations (ODEs), where errors from the unreliable data will be spread across all timescales.

### (b) Using R derived from case data for policy

The growth rate is determined by infectees, but many policies are aimed at infectors. If these groups are different, ignoring this distinction can lead to misapprehensions. For example, a rural area may have R<1 such that cases are entirely driven by incomers from an urban area. If the I(t) in equation (1.1) is dominated by incomers, the R value calculated from equation (1.2) will reflect the R of the urban area and will be unaffected by local measures for the suppression of R.^4^

#### (i) A toy model—two-population SIR

To illustrate the effect of mixing on *R*, we examine a two-population SIR model. Consider an urban population, labelled 1, which lives mainly in a high-R area ($R_{1}=2$), and a rural population, labelled 2, which lives mainly in a low-R area ($R_{2}=0.5$). Both $R_{1}$ and $R_{2}$ follow the normal definition of R within the SIR model based on contact between individuals. For simplicity, we assume the populations are of equal size. The urban population spends some fraction x of its time in the rural area. The model consists of the coupled equations

1.3 $$\frac{di_{1}}{dt}=(1-x)(R_{1}-1)s_{1}i_{1}+x(R_{2}-1)s_{1}i_{2}-i_{1}$$

and

1.4 $$\frac{di_{2}}{dt}=x(R_{1}-1)s_{2}i_{1}+(1-x)(R_{2}-1)s_{2}i_{2}-i_{2},$$

where the populations s,i and r are fractions of the total, and $ds_{1}/dt$ and $dr_{1}/dt$ follow trivially from the terms in $di_{1}/dt$.

**Figure 1.**
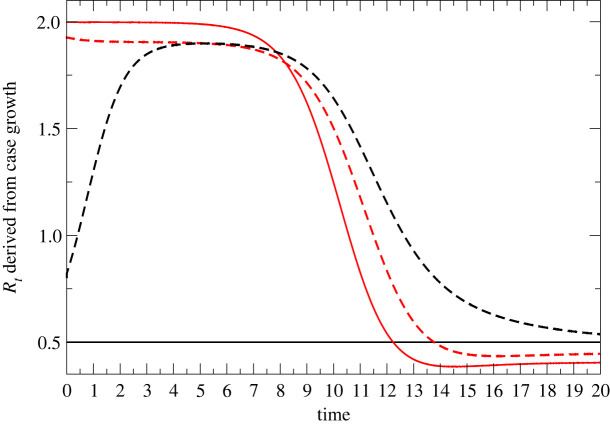
Detectable urban/rural R-numbers from coupled SIR model. Both i(0)=0.00001. Red lines are *R_t_* values derived from urban/rural populations $i_{1}$, and black lines relate to populations $i_{2}$, using equations (1.3) and (1.4). Solid lines show no mixing (x=0); dashed lines correspond to x=0.05. The time axis is in units of the generation time. (Online version in colour.)

Assuming that the measurable quantity is the number of cases i(t), figure 1 shows the results of applying equation (1.1) to infer $R_{t}$. Two cases are considered, as follows.

With x=0, the populations are unmixed: in the urban population, $s_{1}$ is reduced until herd immunity is attained; in the rural population, $s_{2}\approx 1$ and the initial infection dies out exponentially.

A modest 5% mixing is enough to change the picture completely: rural case numbers are driven by incomers, and the measured R-numbers of the two regions become equal.

Although framed in terms of geographical populations, the same principles apply to any coupled subsystems with different levels of infection, e.g. age groups, vaccinated/unvaccinated or isolating/non-isolating. The R-number inferred from cases will always be due to the subpopulation with more cases, not the subpopulation being measured.

The relevance from a policy viewpoint is that measures imposed to suppress $R_{2}$ serve no purpose in suppressing the epidemic, despite the inferred R in that region being well above 1.

Infection rates are also linked to seasonality, and even to weather—contact rates will change if people spend more time indoors. This is a real effect, but weather-induced fluctuations will be of high frequency and indistinguishable from noise. Policy should not depend on previous weather, so ignoring weather as a factor is appropriate.

### (c) R in the UK

Here, we attempt to define and model the R-number in the UK based on contemporary data. We constrain ourselves to using only publicly available data, in particular the case data from the government website [5] and similar data from the Scottish government [6]. Our approach is pragmatic—we do not assume it to be in any sense ‘more accurate’ than in other studies. Specifically, we fit only to case data, not delayed indicators such as hospitalizations and death, so our expectation is that our model will be less accurate than models that use more complete data. Our rationale is that our predictions can be made earlier because lagged indicators are excluded: ‘An 80% right paper before a policy decision is made [it] is worth ten 95% right papers afterwards, provided the methodological limitations imposed by doing it fast are made clear’ [7].

Our model provides R estimates some two weeks ahead of those published by the UK government [5]. To examine whether our method is at least ‘80% right’, we will benchmark our predictions by hindcasting against ‘gold standard’ model-based work contributing to government policy.

In most epidemic theory models, $R_{t}$ is uniquely defined by the rate of growth of the number of infections of the epidemic.

In the UK’s second wave, we had reasonable data for the number of cases as a function of time from positive PCR tests, C(t). This is significantly less than the number of infections as measured by the Zoe and ONS random/weighted [4,8] cohort survey, I(t), but the numbers are proportional, which, as already discussed, is sufficient for R calculation. There is also a delay between infection and test of approximately five days, such that any estimate based on case data will be out of date. We use reports of the first positive test based on PCR by sample date—the ONS cohort survey typically has a larger time between infection and reporting, so is less useful for up-to-date surveillance.

Using case data rather than the cohort study data introduces an important bias, towards a group which has an above-average level of infection. If one imagines that every individual has their own R-number, then the measured R-number is not the average of those individual *R*-numbers. This is because the people with higher individual R-numbers are more likely to be infected, and therefore more likely to be included. As an example, consider two unconnected cities with R-numbers of 1.5 and 0.5—only the first suffers an epidemic and contributes to measured cases. Thus the measured average R-number across the two cities is 1.5.

Another important issue is that because growth is exponential, removing noise using simple averages of R can be misleading. As an example, suppose the true R across two generations is 1, such that the third generation has as many cases as the first. Now, suppose due to noise the measured R values are 2 and 0.5 such that, again, the third generation has the same number of cases. This is all consistent, but applying the average R (1.25) would wrongly suggest a 56% increase. The geometric mean gives a correct result. In general, using arithmetic-averaged R-numbers in place of real noisy data always implies more cases than are present in the data.

## 2. Methods

### (a) Weight, scale and shift methods

The weight–scale–shift (WSS) method [9] is a type of compartmental model in which patients move from one stage of infection to another. Unlike the conventional differential equation approach, in WSS each infection generates an increased population in other compartments in the future. The newly infected population contributes to subsequent compartments via an algorithm in which it is weighted by age group, scaled for probability of moving from one compartment to another and shifted according to the time distribution.

We have previously used such a case-driven kernel compartment model to track the course of the epidemic [9,10]. This work was initiated with a list of cases as a function of time, from either reported historical data or modelled future data.

The simplest case of WSS is a two-compartment model with compartments being the numbers of cases C(t) and deaths D(t). This requires fitting a single kernel relating deaths to cases:

2.1 $$D(t)=\int_{-\infty }^{t}C(t^{\prime })g^{DC}(t-t^{\prime })\;dt^{\prime }$$

in which $g^{DC}(t)$ is the distribution of times between reported cases (i.e. positive tests) and death has been measured from case data and is assumed to follow a lognormal distribution [11]. This can be written as the probability of death on day t, given the number of cases on day $t^{\prime }$: $p(D(t)\;|\;C(t^{\prime }))$. The term $p(D(t)\;|\;C(t^{\prime }))$ incorporates a *scale* factor, namely the case-fatality ratio (CFR), and a *shift* factor by which cases are allocated to deaths at some future time. Using public data for D(t) and C(t), one can infer $g^{DC}(t-t^{\prime })$ from equation (2.1)—alternatively, one could use National Health Service (NHS) data for individual patients to build the distribution. These results agree reasonably well, apart from a spike at t=1, presumably corresponding to people testing positive on their deathbed. This group will probably strongly overrepresent people dying from some other cause and clerical error.^5^

The algorithm proceeds as follows:

(i) At time t, assign new cases to the compartment array representing current cases, C(t).
(ii) These cases are also assigned to an array $C^{\text{old}}(t^{\prime })$ representing the day $t^{\prime }$ on which they move from C(t) to the next stage of infection.
(iii) These cases are also assigned to an array $D_{\text{new}}(t^{\prime })$ (or $R_{\text{new}}(t^{\prime })$) representing the day they move from C to the next stage of infection.
(iv) Newly arrived cases $D_{\text{new}}(t^{\prime })$ and $R_{\text{new}}(t^{\prime })$ are added to the appropriate time in the future.

Not all cases result in death, so $g^{DC}$ is not normalized: recovering patients move into an implicit ‘recovered’ compartment.

The CFR of COVID-19 is strongly dependent on age, so we found it essential to *weight* by age [9,10]. Following the available data, we subdivide the compartments into five-year age bands.

The model can be trivially generalized to multiple compartments, although for each transition the weights, scales and shifts need to be either defined from direct observation of patient data or inferred from time-series observations of the compartments. For UK predictions, we use separate compartments for cases (C), mild (M), illness (I), hospitalization (S), critical care (U), recovering from critical care (V), recovered (R) and dead (D). The full set of equations is given in the appendix.

### (b) Inference from the future and the second law

All processes in WSS are inferred forwards in time from case data. We do not attempt to go backwards in time to infer the infection data (equation (1.1)). The rationale for this is as follows.

There is some distribution of times between infection, symptoms appearing and positive testing, $g^{C|I}$. It may appear that one could apply Bayes’ theorem using the *probability that infection occurred on day t given a positive test on day $t+t^{\prime }$* to infer infections from the case data and the probability of case given infection. However, to do so violates an even more fundamental principle—the second law of thermodynamics, the relevant form of which states that for an irreversible process, entropy (in this case uncertainty about dates) must increase.

A sharp rise in infections I(t) (e.g. from lifting of restrictions) will lead to an increase in C(t) spread across several days. Following the second law, the sharp feature should precede the broad one. We can apply inference *forwards in time* using $g^{C|I}$.

But if we attempt to infer I(t) via projecting C(t) *backwards in time* by applying the distribution of time lags, the features in R will be spread out, giving an implausible situation where sharp features in the case data C(t) arise from slow changes in the incidence I(t):

2.2 $$I(t)=\int_{t}^{\infty }C(t^{\prime })g^{C|I}(t+t^{\prime })\;dt^{\prime }\;\text{is incorrect}.$$

Thus R defined on cases (equation (1.2)) will be more slowly varying than R defined from infections (equation (1.1)).

A similar problem occurs if one tries to infer the time series of cases from death data. In this case, the distribution of times between case report and death is known from hospital records. In the early stages of the pandemic before widespread testing, death data were typically used to infer cases, sometimes erroneously assuming that $g^{C|I}$ and $g^{I|C}$ have the same time dependence and differ only in a scaling factor.

We note that equation (2.2) can be read as ‘Bayes’ theorem cannot be applied backwards in time to an irreversible process’ because of the difference between the concepts of probability distribution and likelihood.

Bayes’ theorem for the probability of a discrete event ‘*C*’ given an event ‘*D*’ is

2.3 $$p^{C|D}=\frac{p^{D|C}P(D)}{P(C)}.$$

When we are dealing with time distribution functions, evolution forwards in time has the form of equation (2.1) if one assumes a flat prior and integrates over all cases. This is fine, because one has no prior information about the future. One can write the analogous equation (2.2), but the assumption of a flat prior is equivalent to assuming the system is initially in a maximum-entropy state, combined with the assumption that the system evolves irreversibly.

The problem lies in assuming that $g^{C|I}(t-t^{\prime })$ is independent of $t^{\prime }$. In some previous work, the ‘reducing entropy’ problem is avoided by using strong low-entropy priors for the infection-based *R*, e.g. insisting that it be piecewise constant [12].

Another way around the causality problem is to assume a model such as SEIR which obeys the second law, and then use Bayesian inference to parametrize the model. However, in this approach, one must assume that the model is correct without evidence from the data.

### (c) What are the case data?

**Figure 2.**
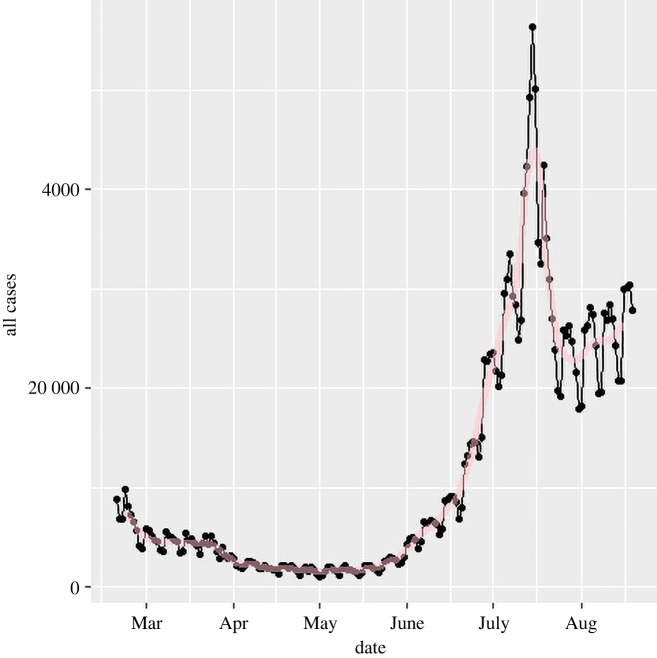
As-published official data on 2021 cases for England (dots). A strong weekly oscillation is evident. Although it is plausible that more infections happen on weekdays when people are at work, we will assume the oscillation is from the amount of testing. The red line shows the effect of applying the seven-day filter (equation (2.5)). (Online version in colour.)

The UK case data (figure 2) consist of daily reports of on the order of $10^{4}$ positive tests. We assume this will be subject to day-to-day statistical stochastic noise,^6^ $\sqrt{C(t)}\approx 100$, and variations in reporting depending on day of the week, so we write the raw data as

2.4 $${\tilde{C}}_{0}(t)=C(t)(1+a(t))+\sqrt{C(t)}\;\eta ,$$

where ${\tilde{C}}_{0}(t)$ is the reported data, defined only at integer t, C(t) is the underlying trend, a(t) is a systematic reporting error and η represents the stochastic noise in the data. Here C(t) is a differentiable function, but η is not. To differentiate this function requires methods from stochastic calculus, but for practical purposes we move directly to using algorithms to deal with the data. In practice, we shall require that the R-number be defined in such a way that if we re-create the epidemic by integrating R through time, it must reproduce the actual epidemic size. We will then see that smoothing the data works best if noise in C(t) is taken to have zero sum, as opposed tonoise in R(t).

### (d) Identifying and eliminating the systematic errors, a(t)

We identify five sources of systematic error in the data:

— false positives and negatives;
— underreporting at weekends, and associated catch-up;
— underreporting on holidays, and associated catch-up;
— delayed reporting at the end of the time series;
— misreporting.^7^

Previously, we estimated the false positive rate to be approximately 0.4% based on CFRs at times of low cases [9]. This was much higher than previously assumed [4] based on the *total* fatality rate in the summer. The cause of this discrepancy may be cross-contamination [13], so the effect is relatively small when the base rate of infection is high. The amount of testing, and by implication the daily number of false positives, has varied relatively slowly compared with the changes in C(t).

We assume this as a constant rate of false positives so that in terms of R-number calculations it has no effect: it simply introduces a constant factor multiplying $\tilde{C}$, which immediately cancels in equation (2.10). In times of high infection, this false positive rate has little effect on total numbers and therefore little effect on further prediction.

Systematic underreporting of cases at the weekend is evident in the data. It is systematic, so we cannot treat it as an enhanced stochastic term. To eliminate this, we make an assumption that across the epidemic the number of infections is independent of the day of the week. Specifically, we rescale the cases by a factor

2.5 $$w_{j}=\frac{\sum_{i}^{7N}\tilde{C_{0}}(i)}{\sum_{i}^{N}7\tilde{C_{0}}(7i+j)},$$

where N is the number of weeks of data. This means that the total number of cases on Mondays is reset to be equal to the total on Sundays, and so on. It removes an obvious source of systematic error.

Across the Christmas period the weekend effect breaks down, and there are even larger fluctuations in the case data. Hence, over the 12-day period from day 153 to 164 (24 December to 4 January), we fit a straight line through the case data, constrained to preserve the total number of cases.

We also investigated a rolling seven-day average. This gives some smoothing, but systematically flattens peaks and fills troughs in the data. The calculation was also repeated by taking seven separate streams of data, one for each day, calculating R based on seven-day changes and then averaging these values.

There is a short delay between positive test and reporting. Using historical data, we found this to be systematic, which allows us to make even more up-to-date measurements. Within Scotland, we find ratios between cases reported for the three most recent days and the final totals for those days. These are 2.9 (±0.2), 1.05 (±0.01) and 1.005 (±0.002), respectively.

The data with these time-dependent systematic errors removed are plotted in figure 10 and denoted by

2.6 $$\tilde{C}(t)=C(t)+\sqrt{C(t)}\;\eta .$$

Henceforth, we will use this $\tilde{C}(t)$ as the case data.

### (e) Stochastic differentials

If we had a differentiable C(t), we could evaluate R as defined in equation (1.2). Unfortunately, the data are $\tilde{C}(t)$, not C(t)—only defined at integer t and with the stochastic noise still present. Nevertheless, we can integrate the equivalent of equation (1.2) and calculate $\tilde{R}$, the ‘R-number with stochastic noise’.

We make a further assumption that $R_{t}$ and τ are slowly varying in time, allowing us to ignore their time-derivatives so that these do not appear in any of the equations.^8^ Integrating equation (1.2), we find that

2.7 $$\int d(\ln\tilde{C}(t))=\frac{(\tilde{R}-1)\Delta t}{\tau }.$$

To calculate the integral, we should use stochastic calculus, and this introduces some ambiguity: case data are available daily, so we can take the discretized form of this equation using the Stratonovitch form,

2.8 $$\tilde{R}(t)=1+\frac{2\tau [\tilde{C}(t)-\tilde{C}(t-1)]}{\left[\tilde{C}(t)+\tilde{C}(t-1)\right]},$$

or its Ito Calculus equivalent,

2.9 $$\tilde{R}(t)=1+\frac{\tau [\tilde{C}(t)-\tilde{C}(t-1)]}{\tilde{C}(t-1)}.$$

Alternatively, we can define $\tilde{R}$ from the exponential form

2.10 $$\tilde{R}(t)=1+\tau \ln\left[\frac{\tilde{C_{1}}(t)}{\tilde{C_{1}}(t-1)}\right].$$

In each case we write $\tilde{R}(t)$, noting that $\tilde{R}(t-\frac{1}{2})$ is more appropriate. All the approaches above were tried, and in terms of final results for R we found little difference between any of these methods. However, if one attempts to regenerate C(t) using these $\tilde{R}(t)$ results by integrating equation (1.2), then only the exponential discretization (2.10) reproduces the time series correctly.

### (f) Estimating the uncertainty in R

However R is calculated, it involves sampling noisy data over some time, during which C(t) itself is varying. Early models assumed that R is constant between changes in policy interventions [12]. If true, this assumption would allow the fitting errors to be calculated precisely, but there is strong evidence that R(t) varies steadily over time due to varying compliance, increased post-infection and post-vaccination immunity and the rise of variants. If R is varying in time, there is a conflict between reducing the stochastic error by sampling over many days and having an up-to-date estimate. We postulate that not only is R(t) differentiable, but also all its derivatives are slowly varying in time. This means that we can reduce uncertainty and make more up-to-date measurements of R by estimating dR/dt and higher derivatives, which is best done using some smoothing function (see §2g).

Since C(t) grows exponentially with R(t), it will be more rapidly varying, and because of variable time from infection to testing, I(t) will vary even more rapidly. The case data actually define a growth rate, which is non-dimensionalized by the generation time τ. Our calculated R−1 is directly proportional to τ, and so when R≫1 probably the largest uncertainty in R comes from the uncertainty of τ. This happens in the earlier stages of each wave of infection. We take a value of τ=5 days [3].

### (g) Smoothing the data

This estimated $\tilde{R}(t)$ has had the systematic errors removed; it is the required R(t) plus a term arising from the stochastic noise.

**Figure 3.**
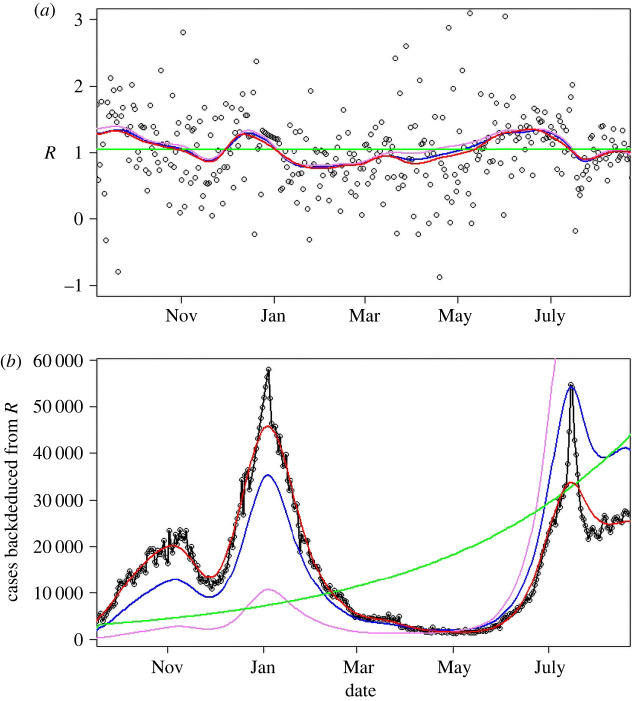
(*a*) R values calculated from various methods in the period September 2020–September 2021. Black circles, $\tilde{R}$ from equation (2.10); violet, Ito integration plus smoothing of $\tilde{R}$; blue, log integration plus smoothing of $\tilde{R}$; red, smoothing of $\tilde{C}$ plus log integration; green, average value. (*b*) Modelled case numbers using these R-numbers from October 2020, with initial case numbers chosen to give correct total number of cases; black circles show actual data, which $\tilde{R}$ reproduces by construction. (Online version in colour.)

Within the UK, daily case numbers were typically of the order of 10 000, so we can expect stochastic noise of $\sqrt{10\;000}$, i.e. about ±1% error in daily growth rate (which is typically of the order of 1%). Thus we can expect that direct calculation of growth from a single day’s change, even with systematic errors removed, will have 100% uncertainty. Figure 3 shows that the noise is indeed dominant, and across the pandemic the standard deviation of $\tilde{R}(t)$ is about 0.6. This value is confounded by the actual root-mean-square variation, $\sqrt{\left\langle {\left(R(t)-\langle R(t)\rangle \right)}^{2}\right\rangle }=0.16$, and by any slowly varying systematic errors such as the effectiveness and amount of testing. We now make our final approximation, smoothing the data to eliminate the high-frequency noise in $\tilde{R}$ while retaining the smoothly varying signal R(t).

Various standard methods of smoothing the data were considered: weekly averaging, LOESS, spline fits with various numbers of splines, and independent spline fits starting and finishing at, or five days after, imposition or removal of lockdown, to account for discontinuity in R(t) when policy changes. Where case numbers are low, the stochastic term is larger relative to the signal, so all fits are weighted by the square root of the number of cases.

All smoothing methods gave similar results, so we chose to use splines and applied them to the various methods of evaluating R: equation (2.9); equation (2.10); mean R across the entire period (1.04); and C(t) from equation (2.6) by smoothing $\tilde{C}$. Figure 3*a* shows that all integration methods appear to give similar variations in R. However, if one attempts to reproduce the trajectory of the case numbers using these different integration measures or spans, small differences in R are magnified (figure 3*b*). This provides further evidence for preferring equation (2.10).

### (h) Do cases rise exponentially?

While R is a well-defined concept in terms of onward infections, the idea of R as a ‘growth rate’ assumes an exponential process. To test whether the data exhibit exponential growth, we consider three models for predicting the case data C(t):

— same as yesterday, C(t)=C(t−1);
— exponential growth, $C(t)=C{\left(t-1\right)}^{2}/C(t-2);$
— linear growth, C(t)=2C(t−1)−C(t−2).

Averaged across all UK regions, we find that ‘same as yesterday’ gives the smallest root-mean-square and mean absolute errors, with linear growth about 1% better than exponential. The effects of noise are significant, but there is no evidence that exponential growth gives the best short-term prediction of growth.

### (i) What value of R causes an epidemic?

In the SIR model, we have exponential growth and any value of $R_{0}$ greater than 1 causes an epidemic in which a finite fraction of the population becomes infected. The ODE approach to SIR assumes complete mixing of the population, but network effects [14–17] can significantly raise the required threshold for $R_{0}$ to cause an epidemic. The exact form of the UK contact network is not known, but there are some well-defined mathematical approximations which can be implemented in an autonome-based model, and it has long been known that allowing spatial variation can affect behaviour in many contexts [18–20].

We simulated a stochastic individual-based model of SIR^9^ with different types of connectivity:

— random connections on a fixed network;
— regular lattices (square, triangular, cubic);
— small-world lattices, with random long-range connections added to a regular lattice.

It is natural to interpret the lattice as a division of people in space, with contact most likely between those living nearby. However, other interpretations of the network are possible; for example, the POLYMOD study [21] shows that contact is primarily with people in one’s own age group.

Each simulation is seeded with 10 infected sites, and S→I or I→R transitions are implemented according to the Gillespie algorithm [22]. Once the network is defined, this model has only one parameter, $R_{0}$, the ratio of attempted infection rates^10^ to recovery rate.

**Figure 4.**
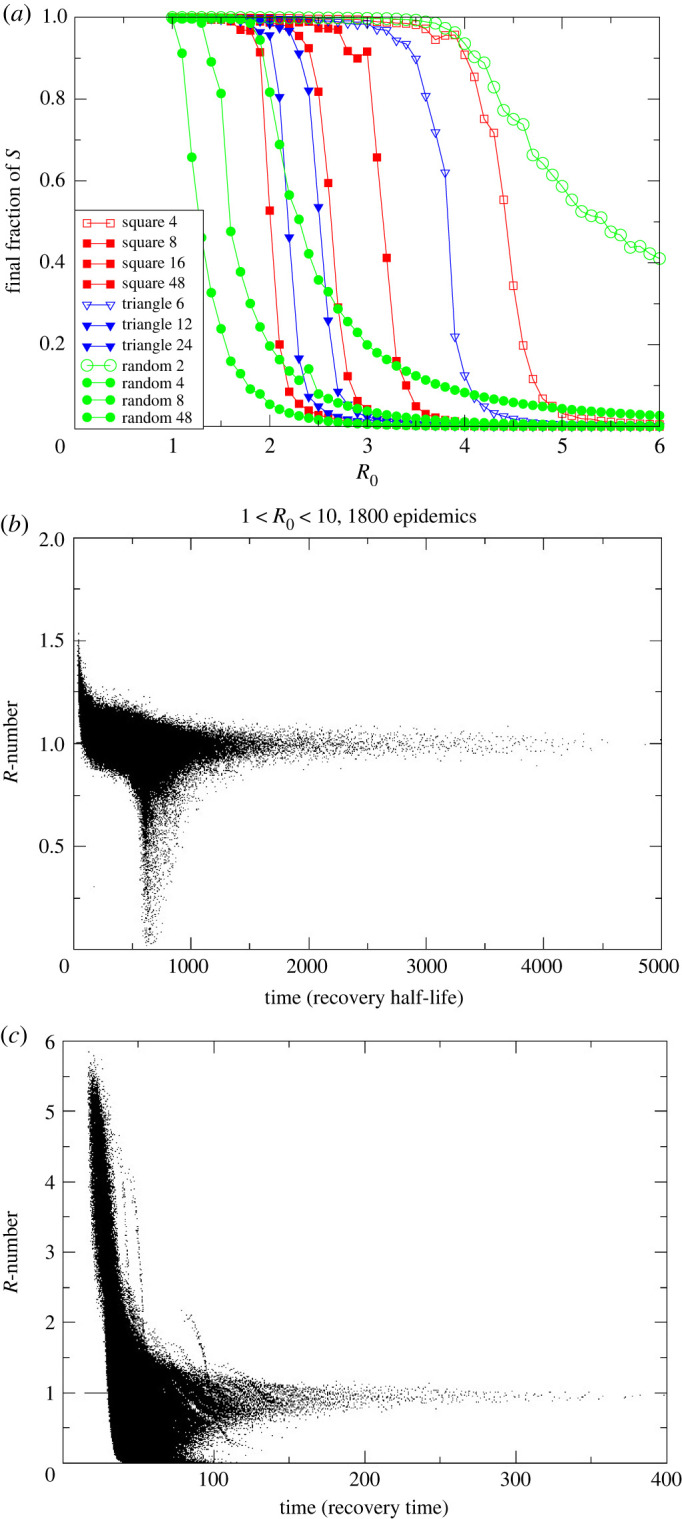
(*a*) Size of the final epidemic for various network structures and values of $R_{0}$. Legend gives the different lattice structures and the number of connections each has. To set equivalent $R_{0}$, infection probability per link is lower in more highly connected lattices. (*b*) Scatterplot of measured R(t)=−ΔS/ΔR from simulations with eight-neighbour square lattice, 500 000 sites, and $R_{0}$ ranging from 1 to 10. Twenty simulations at each 0.1 increment in $R_{0}$ are shown. Timescale has recovery rate set to 1 and R(t) is plotted against t in units of the recovery time. Other lattices are similar. (*c*) Small-world version of (*b*) with eight neighbours plus one added long-range connection. (Online version in colour.)

It is evident from figure 4 that $R_{0}=1$ is a poor predictor of whether the infection triggers an epidemic. The ODE result of a threshold at $R_{0}=1$ is recovered for a fully connected network. Less densely connected random networks require dozens of connections per node to generate an epidemic with $R_{0}=1$. For sparser networks, the total number of infections can be significantly less than the total population. For two-dimensional lattice networks, the threshold for an epidemic is $R_{0}=2$. This can be understood by noting that the SIR lattice can be mapped to a reaction–diffusion equation, which generates a travelling wave [23] moving at constant velocity—in the SIR context autonomes behind the wave are predominantly I and R, while ahead of the wave they are S. New infectees typically lie on the boundary between previously infected and fully susceptible regions—so compared with the early transient only approximately half as many neighbours are S.

The R-number for these lattice models is shown in figure 4*b*,*c*. These scatterplots come from many hundreds of different simulated epidemics with an order of magnitude variation in $1<R_{0}<10$. Each point represents the value of $R_{t}$ which would be measured in the epidemic. Individual epidemics are not tracked, but two distinct behaviours are evident: either the epidemic does not spread and $R_{t}$ drops to zero after some time, or it does spread across the system.

Remarkably, for any $R_{0}$ large enough to generate an epidemic, $R_{t}$ tends to 1 after some transient time. This behaviour is completely different from that of an ODE-based well-mixed SIR model, for which the value of $R_{t}$ decreases steadily over time with no special behaviour as it passes through 1 (figure 1).

Epidemics spread throughout the system on the two-dimensional lattice only for $R_{0}>2$, and a much higher value is required for two-dimensional networks with fewer connections. The epidemic on a network goes through the following two distinct stages.

— First, there is exponential growth, with R(t) dropping with time for a transient period.

Then:

— *either* R(t) drops below 1 and the epidemic dies without spreading;
— *or* a wavefront is established and R(t) drops to 1, and the epidemic spreads in wave-like fashion through the system.

It is debatable whether R(t) is a useful quantity for non-exponential growth, but it is still readily definable and measurable from equations (1.1) and (1.2).

The lattice model neglects long-range connections: we introduce these with a ‘small world’ network in which additional random connections to anywhere in the system are added to the eight neighbours. The R-numbers for such a network with one long-range connection per site are depicted in figure 4*c*. The plot is broadly similar to figure 4*b*, although note the 10-fold difference in the time axis. We see that:

— the timescale of the epidemic is very much reduced by the long-range connections;
— the high-$R_{0}$ epidemics retain a high value of R, because the epidemic has spread through the entire system before the transient ends;
— intermediate values of $R_{0}$ cause epidemics but still tend to R(t)=1.

We observe that the dimensionality of the network is important and distinct from connectivity; for example, in a one-dimensional ‘line’ of autonomes with local connection the infection can never spread through an infinite system, regardless of how many neighbours are connected.

## 3. Validation

### (a) Sensitivity of R to fitting methods

**Figure 5.**
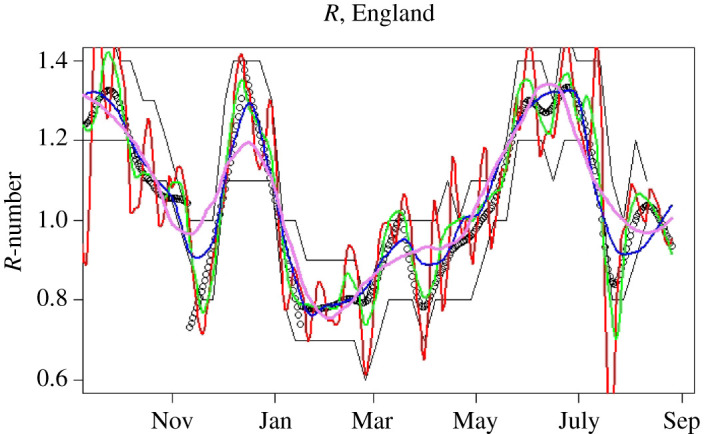
R-numbers for England, September 2020–September 2021. Black points represent our central estimates, based on piecewise fits between major locking and unlocking events. Red, green, blue and violet lines show LOESS smoothed R-numbers from equation (2.10) with span=0.05, 0.1, 0.2 and 0.3, respectively (span controls the amount of smoothing). Black lines are the published bounds on R data from the SPI-M consensus; to obtain this agreement, the consensus values are assigned to a date 16 days before publication. (Online version in colour.)

In addition to the type of smoothing applied, the amount of smoothing leads to variations in predicted *R*. Figure 5 shows independent piecewise fits to periods between lockdowns and unlockings. Curiously, the discontinuous piecewise fits are found to still give nearly continuous behaviour, the one exception being around Christmas 2020 and New Year 2021 where the reporting data are erratic and do not follow the weekly variations. So, we can reasonably assume that R(t) is a slowly varying function and that dR/dt is a continuous function which can be used to improve the estimate of R(t) beyond the average over the smoothing period and into the future. All of these features mean that the uncertainty in our R(t) will be much lower than the residuals typically calculated by fitting codes, although without knowing exact functional forms it is impossible to know by how much.

A final check on the uncertainties in the method comes from comparing the R values obtained by the different smoothing methods and different smoothing periods (figure 5). Reassuringly, these are all consistent within ±0.1.

### (b) Validation by reverse-engineering the epidemic

Since the R-number is the gradient of the case numbers, it should be possible to recreate the case number data using only the R-number and the initial caseload. If $\tilde{C}(t)$ were a continuous variable, this would be straightforward, but if we smooth $\tilde{C}(t)$ or $\tilde{R}(t)$, then we lose information because the smoothing process is not reversible.

Figure 3 compares the actual number of cases with those regenerated from R-numbers. One sensible constraint is that, whatever we do, the total number of cases should be correct, i.e.

$$\int C_{0}\exp\left[\frac{(R(t)-1)t}{\tau }\right]\;dt=\int \tilde{C}(t)\;dt,$$

where the left-hand side is the modelling cases and the right-hand side the data. This is done by adjusting $C_{0}$, the initial number of cases, which allows re-created trajectories from different smoothing methods to be compared on an equal footing.

Using $\tilde{R}(t)$ exactly reproduces the data, but all smoothed versions of $\tilde{R}(t)$ overestimate the growth rate; because the curves shown are adjusted to give the correct total number of cases, this manifests as the second peak being much higher than the first. Smoothing the case data first and then calculating R from the smoothed case data gives a better fit, with the feature somewhat broadened for reasons similar to those given in §2b.

We note that the error arises in part because *the form of the noise is not known*. So, for example, if we assume a form for η such as white noise or Gaussian random variable such that

∫η(t) dt=0,

then it trivially follows that

∫exp⁡(η(t)) dt≠0.

Since the R-number appears in the exponential of the epidemic growth, it follows that the ‘noise’ makes a non-zero contribution to the growth rate, which should or should not be incorporated in R(t) depending on R’s precise definition.

We see that stochastic integration using Ito’s method gives the worst results, leading to a systematic overestimate of R which equates to too-high case numbers at long times. Integration using log cases performs better. The better reproduction of the epidemic (figure 3) suggests that it is better to treat the noise in $\tilde{C}$ rather than $\tilde{R}$.

### (c) Validation by appeal to authority

The value of R is not directly measurable, so there is no way to empirically validate these results. We therefore compare our predictions with those from more sophisticated epidemic models from the UK government’s SPI-M [24] committee.^11^ UK Government data about R are derived from a weekly consensus across many different methodologies and groups.^12^

It is clear from the figure that our R estimates are compatible with the reference values published 16 days later. This is reasonable, since published data are stated as being averages over the preceding weeks. The SPI-M consensus is reached during the week prior to publication in advance of the published data and the values are therefore available to policymakers earlier. Nevertheless, our direct method is capable of providing equivalent values well in advance of the currently published values.

A definitive empirical measurement of R is lacking, so it is possible that both simple and detailed models are similarly wrong. Regardless, our method has been demonstrated to be an excellent predictor of future published results.

## 4. Implementation and results

### (a) Code and subdivisions

The R calculation is implemented by the WSS [25] codebase, which is publicly available and written in the statistical programming language R. WSS uses imported case data updated daily, and produces estimates of subsequent hospitalizations, deaths and recoveries. It executes within minutes on a single processor.

**Figure 6.**
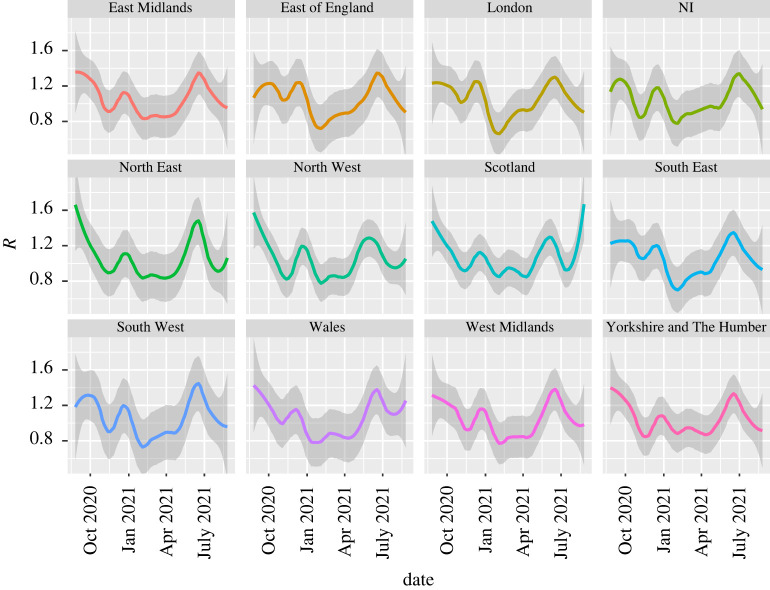
R-numbers for UK nations and English regions calculated with WSS; shading represents the LOESS confidence interval associated with the smoothing (here, LOESS with span=0.3). December and June peaks associated with the Alpha and Delta variants are evident in all regions. Blips in September and March correspond to low case numbers and may be artefacts. (Online version in colour.)

The WSS code generates R-numbers at the regional level (figure 6). The statistics for the four UK nations and nine English regions are sufficiently good to produce stable independent estimates at that resolution, and are consistent with the SPI-M published values (subject to 16-day lags). We also evaluated R at the level of individual health boards in Scotland. These values showed plausible trends, except for the smallest boards. The issue there is due to not only insufficent data, but also the fact that rural values may be driven by incomers as already discussed in §1b. Consequently, case data may not be indicative of community transmission rates in those areas. The local authority regions in England also often have too-small numbers for accurate evaluation, although a combination of large R and high case numbers can be indicative of local hotspots or superspreading events.

**Figure 7.**
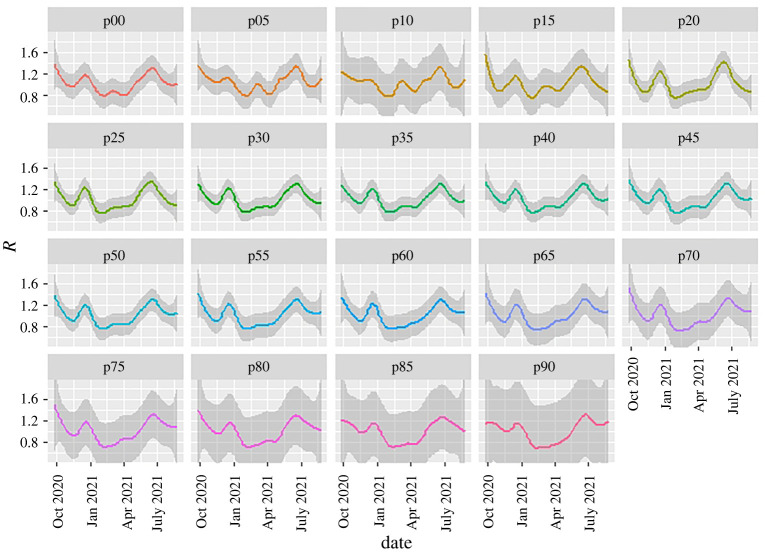
WSS R-number prediction method applied to case data split by five-year age groups, with ‘p’ indicating the youngest year-group. Data are averaged across all England; shading represents the LOESS confidence interval associated with the smoothing (here, LOESS with span=0.3). Uncertainty increases by age because of larger fluctuations, which in turn arise from smaller total numbers of cases. See main text explaining why this is a scaled growth rate and not a conventional R-number. (Online version in colour.)

The data can also be sliced to provide a growth-rate breakdown by age group (figure 7). Breakdown by age has a similar problem to that for regions because the case data refer to infectees, not infectors—and generally infectors are in a different age group from infectees [21]. This intergenerational mixing is particularly true for families, hospitals and care-home situations. Specifically, when case numbers are unevenly distributed across age groups, the ‘R-numbers’ ascribed to older age groups do not imply that these people are responsible for infection.

### (b) Vaccination effects

Vaccination is known to reduce transmissibility of the virus by 60–90% [26–33]. It may seem mysterious that there is little sign of an effect of vaccination in the national or regional *R-*numbers. To understand this, one needs to look more deeply into the data. Figure 7, the ‘R-number’ sliced by age group, shows the large reduction in R for the older age groups during the vaccination roll-out (late 2020 to early 2021) as case numbers are suppressed. Infection preferentially shifted to the unvaccinated age groups, and our overall R-number is weighted across subgroups by cases, not population. So the national R is dominated by the younger population.

Furthermore, R represents the rate of increase in infections, not the total numbers. Thus it is affected only by the rate of increase of vaccination, not the total numbers. We see that R in the older age groups in July rebounded to the national average once almost everyone in those groups had been vaccinated. However, the case numbers in the older age groups remain low thanks to the strong suppression of R during the vaccine roll-out. This similar R across age groups implies that they are mixing. Given the relative prevalences, it represents infection of the older age groups by the younger, unvaccinated population, rather than transmission within one age band, similar to the situation for the urban/rural model (figure 1).

### (c) Detection of events

It is possible to detect individual events in the data and to test correlations by investigating appropriate subdivisions. For example, the peak in December 2020 associated with the Alpha variant can be seen to occur earliest in the South East and later further north, consistent with its believed origins in Kent. Conversely, the June 2021 peak associated with the Delta variant appears first in the North West, then almost simultaneously everywhere else, suggesting multiple importations rather than geographical spread.

**Figure 8.**
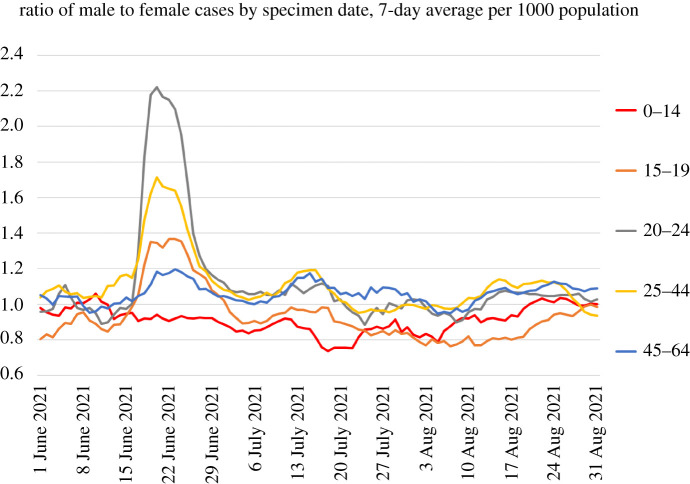
Ratio of male to female COVID-19 cases by age group, based on UK government data [5], showing a sharp peak in June 2021. The peak is similar to the two-population behaviour of figure 1. (Online version in colour.)

Increases correlated with reopening of schools can be seen to occur first in the youngest age groups and the typical age groups of parents, again strongly suggestive of causation. Furthermore, the peak in July, which has been associated with sporting events such as the European football championship final, can be seen to be initially driven by men and spreading subsequently to women. Data from event attendance were equivocal [34], but the case data are striking (figure 8).

### (d) Features beyond R

The WSS approach can be applied not only to I(t) and C(t), but also to any other quantity, for example hospitalization or death rates. Unlike conventional ODE-driven compartment models, WSS incorporates a delay moving from one compartment to the next; thus cases are related to deaths via a generalization of equation (2.1),

4.1 $$D_{\text{WSS}}(t)=\int_{-\infty }^{t}C(t-t^{\prime })g^{D|C}(t,t-t^{\prime })\;dt^{\prime },$$

where $g^{D|C}$ is the probability of death at time t given a case reported at time $t^{\prime }$. Note that the forwards projection avoids the entropy-decrease problem discussed in §2b, correctly predicting that sharp peaks in C(t) will lead to broader peaks in D(t).

We write $g^{D|C}(t,t-t^{\prime })$ as a function of two variables. The $t-t^{\prime }$ dependence represents the trajectory of the illness from infection to death; this has been determined in clinical studies. The t dependence represents changes in disease severity over time. Disentanglng these, we can write

4.2 $$D_{\text{obs}}(t)=g_{0}^{DC}(t)\cdot D_{\text{WSS}}(t)=g_{0}^{DC}(t)\int_{-\infty }^{t}C(t-t^{\prime })g_{1}^{D|C}(t-t^{\prime })\;dt^{\prime }.$$

This $g_{0}^{DC}(t)$ is a time-dependent CFR. The delay between case and death means one cannot simply use C(t)/D(t): $g_{1}^{D|C}$ provides the shift forward in time from case to death; it is represented by a gamma distribution, normalized and fitted to case and death data across the entire pandemic.^13^

**Figure 9.**
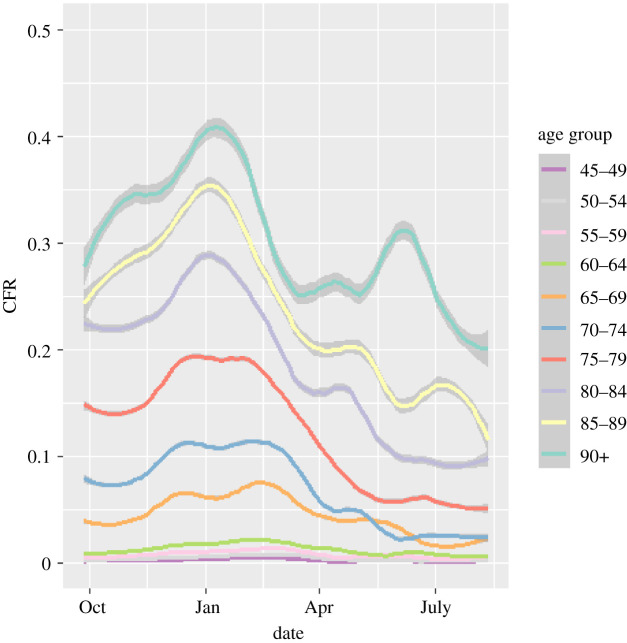
Case-fatality ratios, 2020–2021, plotted as deaths per case from the WSS model, by age group. Lines are weighted smoothed fits to the data. CFR graphs for people aged under 45 are excluded as they are so low. Shading shows uncertainty introduced by smoothing day-to-day variations, excluding errors on the mean from small-number statistics in September 2020 and May 2021. The eye-catching peak for the 90+ age group in June 2021 is probably a small-number effect, and it can be eliminated completely by combining the 85–89 and 90+ age groups. (Online version in colour.)

The function $g_{0}^{DC}$ is dependent on age group; we use separate functions for each five-year age band. If the lethality of the infection had remained constant throughout the epidemic, then $g_{0}(t)$ would be constant. If fact, its changes as shown in figure 9 provide a powerful image of the changing lethality of the epidemic. There are three salient features.

**Figure 10.**
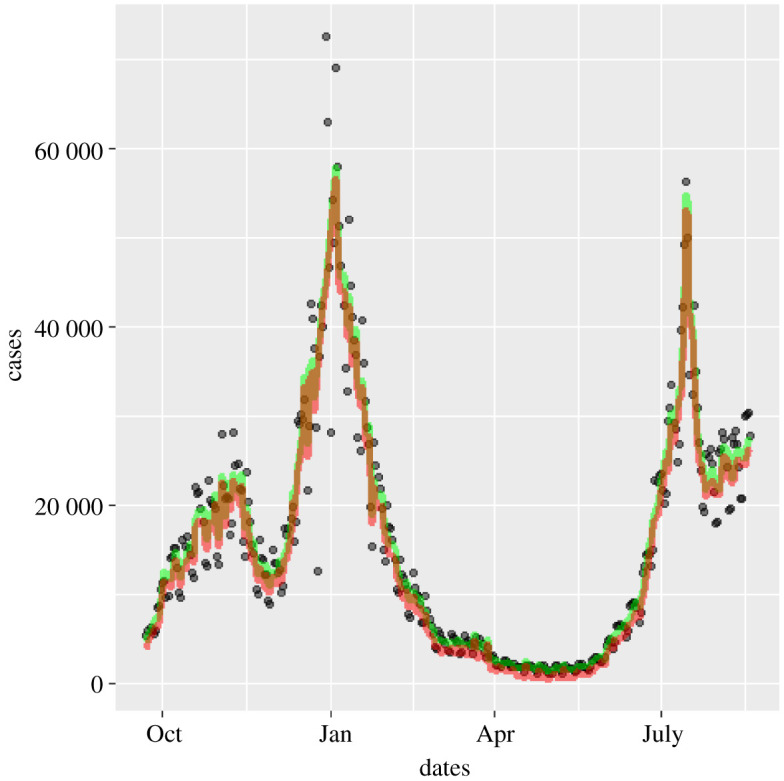
Effect of removing weekend and Christmas systematic errors on 2020–2021 cases: positive first PCR test data as published (circles) [5], weekend and Christmas smoothed case data (green) and data corrected for 0.4% false positives (red) [10]. (Online version in colour.)

**Figure 11.**
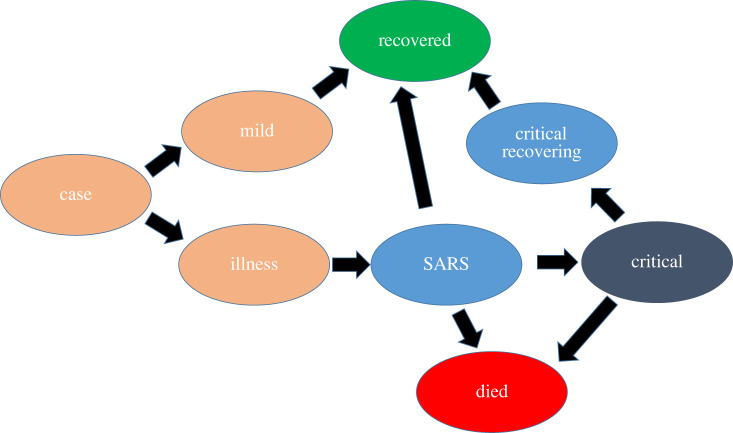
Schematic flow chart for WSS compartment model. The SARS compartment acts as a proxy for hospitalization, and the critical compartment for intensive care. (Online version in colour.)

The Alpha variant was accompanied by a pronounced increase in the CFR $g_{0}^{DC}(t)$, in all age groups and regions through December, plateauing once Alpha became ubiquitous by 2021 [9]. The sharp decline in 2021, and the onset of the effect in the oldest age group first, can be associated with the effect of the vaccine on causing milder infections. The age-dependence of CFR is so pronounced that for the under-45 population (not shown) statistics are too poor for reasonable analysis.

A discernable blip in the drop of the CFR during May 2021 could be associated with the arrival of the Delta variant (figure 9).

As well as age-related factors, WSS enables us to discern differences in the CFR across different geographical regions. This indicates a strong north–south divide: the CFR is significantly lower in the south except for a short window when the Alpha variant was more prevalent there. Results have been discussed in previous work [9,10] and are not repeated here.

The $g_{0}^{DC}(t)$ function gives rapid, real-time monitoring of the epidemic, which allowed WSS to provide the first published evidence for increased lethality of the Alpha variant, as well as the effects of the Delta variant and vaccination [9,10].

## 5. Conclusion

The published R-number from SPI-M can be predicted some 16 days in advance of publication by statistical analysis of the publicly available case data using our WSS code.

Our case data estimates are themselves necessarily delayed by the time between infection and positive test, so it is likely that the published values are delayed by around three weeks from the actual spreading events. It is likely that the SPI-M modelling will provide more reliable estimates; however, our WSS model appears to be adequate for making coarse policy decisions. For some applications, the earlier availability is likely to outweigh the loss of accuracy.

The case number-based R smooths any sharp discontinuities in infection-based R. WSS is probably less reliable as a tool for analysing the effects of non-pharmaceutical interventions (NPIs) than models which incorporate infections explicitly and are parametrized using a Bayesian approach. However, the WSS R allows us to create a narrative of the second wave:

— an increase of cases through September and October, with R>1;
— a sharp reduction of R with NPIs in November 2020, rebounding as the B1.117 (Alpha) variant became established;
— a sharp drop of R at the January lockdown (the case data show a continuous drop, but this is consistent with a sharp drop in infections after 6 January, smoothed by variable incubation times);
— a steady rise in R throughout February and May, accelerating as the B1.617 (Delta) variant became established and restrictions were released;
— a sharp peak and drop in R in July, despite relaxation of restrictions;
— a rise in R in Scotland during August, not mirrored in England.

The WSS code also produces up-to-date CFRs [10]. Analysis of these in figure 9 reveals a sharp decrease in CFRs correlated with the vaccine roll-out, showing that vaccination has a double benefit of reducing infections and ameliorating the effects of COVID-19. The reduction in the CFR is about 50%, and this has continued during the rise of the B1.617 variant. Correlation does not imply causation, but a protective effective of vaccination seems more likely than other possibilities consistent with the data, such as B1.617 being less deadly than B1.117.

We note that it may seem counterintuitive that R is increasing during the vaccination programme. This is because R derived from case data is not the average over the population, but rather the average over those who are infected. Eliminating infection from a vaccinated subpopulation would mean that the reported R refers only to the unvaccinated population. Perhaps the most surprising outcome of this study is the excellent agreement of the results of this simple method with those of far more detailed epidemiological models. This indicates that the case data currently being produced are sufficient to track the trajectory of the epidemic.

The R-number is well defined but unmeasurable in terms of who infected whom. It can be inferred from case data, but its relation to the growth rate rests on the assumption of short-term exponential growth with slowly varying *R*. This follows from a well-mixed ODE implementation of SIR or related models, whereas a lattice-based implementation of SIR gives linear growth. These are limiting cases of a range of network models. The data from the UK coronavirus epidemic have features closer to the lattice-model end of the spectrum. The R-number has remained close to 1, with external shocks such as variants producing transient peaks in R of a few weeks’ duration before returning to 1. This happened both with a lockdown in January and without one in July. Similarly, the epidemic is more reliably reproduced from R-numbers derived from smoothed cases, rather than smoothing the R-number itself. This indicates that short-term fluctuations in case data are additive rather than multiplicative, implying medium-term linear growth rather than exponential growth.

The effects of lockdowns and other measures in reducing cases and suppressing spread are significant in all cases—in a well-mixed model this manifests as a lowered herd-immunity threshold, and in the lattice models as a slower-moving wavefront. Long-distance travel bans have the effect of reducing long-range connections, making the network more lattice-like.

The lattice model indicates that an initial value of $R_{0}$ above 2 is required to generate a sustained epidemic, as opposed to 1 for a well-mixed model. However, if the disease spreads as a wave, it generates slightly higher total case numbers than in the well-mixed case. We note that an *observed* R(t)=1 value is consistent with a much higher $R_{0}$, and that significant reduction of $R_{0}$ may have little effect on R(t): individuals at the wavefront can only become infected once, even when a high $R_{0}$ implies they may have several encounters which could lead toinfection.

Medium-term epidemic predictions for hospital occupation, ICU demand and deaths are extremely sensitive to assumptions regarding R(t). Whereas WSS assumes that R(t) will return to 1 after a transient event, SPI-MO has produced ‘scenarios’ based on the assumption of fixed R(t), and the assumption that it is fixed has a bigger effect than the value chosen. As deduced from the UK case data, R(t) has remained close to 1, with occasional excursions producing short-lived transients. The Omicron variant had an $R_{0}$ of around 3 [35], but R(t) returned to 1 within a few weeks. The Alpha variant, originally detected in Kent, behaved similarly, although national peaks are broadened as it spread geographically from south to north in a couple of months. It appears that in the UK the coronavirus has spread on a network dominated by localized interactions. WSS has been used for weekly nowcasts and medium-term predictions as part of a suite of codes by SPI-MO and the UK Heath Security Agency. This has demonstrated that predictions made using only case data can produce timely results with accuracy indistinguishable from that of more sophisticated models.

## Data Availability

The WSS code is written in the statistical programming language R and is available at https://github.com/gjackland/WSS. It has been under continuous and ongoing development throughout the pandemic. Data sources are accessed automatically at runtime from web sources with URLs embedded in the code. The WSS model was used by the Scottish Government and the UK Joint Biosecurity Centre for the ensemble of R-number estimates from June 2021, and the full WSS model contributed to the SPI-MO weekly consensus report and predictions of deaths and hospitalizations across the UK nations and seven English regions from November 2021.

## Footnotes

1 In which case it is equivalent to growth rate.

2 For example, it could be modelled as a reaction–diffusion process in two dimensions, for which cases grow quadratically in time.

3 Another term used ambiguously in the literature.

4 Changes in calculated R will exhibit some delay from the exponential decay of R<1, and local suppression measures will speed this decay.

5 The NHS line list includes approximately 1.5% of deaths reported before test.

6 This assumes independent infection events; if superspreading events are significant the noise will be larger, although some smoothing is already provided by the distribution of times from infection to test.

7 The reported number of cases for England is approximately 1% higher than the sum of the regions; this is because of cases which cannot be assigned to any region.

8 A counterexample might be that the generation time would reduce if people self-isolate after a positive test, so onward transmission only happens early in the infectious period [1].

9 This is the obvious stochastic cellular automaton generalization of SIR. We first coded it for the DAP computer installed at EPCC 1984, when it was already a well-established demonstration application for SIMD architecture.

10 Which fail if the target is already in state I or R.

11 https://www.gov.uk/government/groups/scientific-pandemic-influenza-subgroup-on-modelling.

12 https://www.gov.uk/government/publications/reproduction-number-r-and-growth-rate-methodology/reproduction-number-r-and-growth-rate-methodology.

13 In keeping with the spirit of WSS, we used publicly available data, but since the original submission, we have been able to check this against confidential data on case–death intervals for individual patients.

## Appendix A

**(a) Vanilla weight–scale–shift model**

WSS is a compartmental model with transitions through compartments driven by lognormal time kernels.

There are eight compartments, taken from the CovidSim model: cases (C), mild (M), illness (I), hospitalization (S), critical care (U), recovering from critical care (V), recovered (R) and dead (D); the letters in brackets represent the number of people in each compartment and vary with time. Because the case data are available daily, these are defined only at integer values of *t*. There are separate compartments for each five-year age group.

Transition is only allowed between certain compartments as depicted in figure 11.

The code is ‘driven’ by cases. WSS has no infection model and therefore no capability to predict future infections or effects of policy. However, such effects can be retrospectively determined because C(t) contains information about R-numbers.

At time t, we use the kernels to increment the numbers which will leave the compartment at a future time $t+t^{\prime }$.

Values for C(t) are read in from data.coronavirus.gov.uk with a seven-day oscillation filter. The model equations are as follows:

A1 $$\begin{array}{rl} & M(t)=\int_{\tau =1}^{t}g^{MC}(t-\tau )C(\tau )-g^{RM}(t-\tau )M(\tau )\;d\tau , \end{array}$$

A2 $$\begin{array}{rl} & I(t)=\int_{\tau =1}^{t}g^{IC}(t-\tau )C(\tau )-g^{SI}(t-\tau )-g^{RI}(t-\tau )I(\tau )\;d\tau , \end{array}$$

A3 $$\begin{array}{rl} & S(t)=\int_{\tau =1}^{t}g^{SI}(t-\tau )I(\tau )-g^{DS}(t-\tau )S(\tau )-g^{RS}(t-\tau )S(\tau )-g^{US}(t-\tau )S(\tau )\;d\tau , \end{array}$$

A4 $$\begin{array}{rl} & U(t)=\int_{\tau =1}^{t}g^{US}(t-\tau )S(\tau )-g^{VU}(t-\tau )U(\tau )-g^{DU}(t-\tau )U(\tau )\;d\tau , \end{array}$$

A5 $$\begin{array}{rl} & V(t)=\int_{\tau =1}^{t}g^{VU}(t-\tau )U(\tau )-g^{VR}(t-\tau )V(\tau )\;d\tau , \end{array}$$

A6 $$\begin{array}{rl} & R(t)=\int_{\tau =1}^{t}g^{RU}(t-\tau )U(\tau )+g^{RS}(t-\tau )S(\tau )+g^{RI}(t-\tau )I(\tau )+g^{RM}(t-\tau )M(\tau )\;d\tau \end{array}$$

A7 $$\begin{array}{rl} \;\mathrm{and}\; & D(t)=\int_{\tau =1}^{t}g^{DV}(t-\tau )V(\tau )+g^{DS}(t-\tau )S(\tau )+g^{DI}(t-\tau )I(\tau )\;d\tau , \end{array}$$

where $g^{ij}(x)$ is the probability of transferring from compartment j to compartment i after some time x. These are the fitting parameters of the model. To make the model tractable, we break them into normalized lognormal time distributions and age-dependent total probabilities, so that each transition is characterized by three numbers: probability, mean and width of the lognormal distribution.

These parameters are fitted to observed death, prevalence, hospitalization and ICU occupation data from across the whole pandemic. Key to this are the ratios between the compartments, i.e. the CFRs.

**(b) Application modes of weight, scale and shift**

WSS can be run for three distinct purposes, depending on what is regarded as an input or a parameter and what is an output. In the most general case, the various g’s are distribution functions which may change over time. For simplicity, we split each function into scale and shift terms,

$$g(t,\tau )=g_{0}(t)g_{1}(\tau ),$$

where $g_{1}$ is a lognormal probability distribution function defined by two parameters and $g_{0}$ is the fraction of people transferring between compartments. The calculation of R-numbers is independent of the g’s. The weighting by age group is applied at all times.

**(i) Nowcasting**

In nowcasting mode, we fit time-independent g’s and run the WSS model to reproduce the epidemic data, including deaths, hospitalizations, ICU occupation rates etc. The fit can be done over the entire pandemic or, if medium-term predictions are required, only over more recent data. The main purpose of this oversimplified model is to calculate R and the growth rate, which are independent of the g’s.

**(ii) Monitoring and variant detection**

In this mode, we use the full daily datastreams for occupation of compartments i and j. We can calculate the $g^{ij}(t)$ required at each day to precisely reproduce the i and j datastreams. The change in this fitted g(t) can be used to monitor changes in the behaviour of the epidemic. For hospital data, this information was not available by age group, so we used an age-independent time-dependent rescaling factor across all age groups, applied to an age-dependent constant base rate.

Since October 2020, the vanilla WSS model has failed to track the published death data—we attribute this failure to the variants Alpha, Delta and Omicron and to vaccines. These are treated using the $g_{0}$’s—we do not separate compartments by vaccine or variant status.

Treatments such as dexamethasone were already in place before the model starts in August 2020, so are already factored in. We do not explicitly model new antiviral medications, but their effect is in part captured by the vaccine effect as they were rolled out in the same period.

**(iii) Hindcasting**

The monitoring reveals that changes in the g’s come from variants and reduced severity due to vaccination and prior infection. Consequently, we employ a parametrized model for $g_{0}(t):$

$$g_{0}(t)=\sum_{\alpha ,\nu }g_{\alpha ,\nu }f_{\alpha ,\nu }(t),$$

where α and ν label variant and immunity status, each of which has a different constant $g_{\alpha ,\nu }$. The time dependence of $g_{0}(t)$ now comes entirely from the proportion of variants and immunity in each age group and area, for which time-series data are available online [4,5]. With this data-driven time dependency, we have a model for the full trajectory of the epidemic based only on case data.

**(iv) Forecasting**

In forecasting mode, we weight the fitting of the $g_{0}(t)$ and the modelled occupations of each compartment to the most recent data. Predicted vaccination effects are included, and in periods of transition between variants the $f_{\alpha ,\nu }$ follow a logistic curve. We then predict the R-number into the future to generate future case data and run the WSS model. In the short term, predictions depend mainly on known case data, but beyond four weeks the forecasts depend almost entirely on predicted case numbers, which in turn depend on our predicted R-numbers. Changes in $g_{0}(t)$ due to variants and vaccines are incorporated into these models.

A feature of the case-driven approach is that we do not attempt to measure infection probabilities. Thus the parameter fitting is optimized to describe disease outcomes.

**(c) Incidence and prevalence**

WSS obtains incidence from the number of cases (which is an input). This is multiplied by a scaling factor representing how many people are infected but never tested, the factor being obtained from the ONS incidence estimates [4]. This factor appears to lie between 2 and 3. Prevalence is then the number of people in all compartments, again multiplied by the missing incidence factor.

Initially, prevalence and incidence are outputs of the model and do not feed back into the dynamics except via the immunity factors as an additional ‘type’ of vaccine. This had little effect on predictions until the Omicron outbreak. Here, the combination of waning vaccine immunity and large case numbers meant that previous infection with the Omicron variant made a large contribution to overall immunity.
